# Response of tef (Eragrostis tef (Zucc.) Trotter) yield to nutrient management under rainy and irrigation production systems in northwestern Amhara, Ethiopia

**DOI:** 10.1371/journal.pone.0315730

**Published:** 2025-01-31

**Authors:** Zerfu Bazie, Tadele Amare, Erkihun Alemu, Bitewulign Kerebh, Abere Tenagn, Abrham Awoke, Zmie Ambaw, Beamlaku Alemayehu, Anteneh Abewa, Ataklte Abebe, Tesfaye Feyisa, Birhanu Agumas

**Affiliations:** 1 Adet Agricultural Research Center, Adet, Ethiopia; 2 Gondar Agricultural Research Center, Gondar, Ethiopia; 3 Amhara Agricultural Research Institute (ARARI), Bahir Dar, Ethiopia; Osmania University, INDIA

## Abstract

Crops respond differently to soil nutrients because of climate, soil, and management. This study aimed to determine the most important nutrients for tef production. The experiment was conducted over two production seasons. All (NPKSZnB), All-B, All-Zn, All-S, All-K, All-P, All-N, RNP, RNP+Sx1, and no fertilizer treatments were applied. The pre-planning soil status of the study sites, available P concentrations during the rainy season are between 5.1 and 8.9 mg kg^-1^, however, 21.4 mg kg^-1^ of available P is observed during irrigation. In rain-fed production systems, the mean soil N concentrations are 0.12% in nitisols and 0.15% in vertosols, whereas in irrigation production systems, it is 0.14% in nitiosols. The grain yield significantly (p ≤ 0.01) varied with the omission of nutrients during the production season. However, there was no significant (p<0.05) decrease in yield due to the omission of KSZnB nutrients. The lowest mean grain yields of 342 kg ha^-1^ (nitisols) and 491 kg ha^-1^ (vertosols) were obtained from the no fertilizer treatment. Nitrogen omission decreased yields by 49 and 65% in nitisols and vertosols under the rainy season, respectively, whereas a 19% yield decrease was also observed in the irrigation season. A 10% yield decrease was observed from the P omitted treatment in the rainy season. Thus, N is the primary limiting nutrient to yield in both production seasons, while P is also a yield-limiting nutrient in nitisols. Thus, the government should import the right kind of fertilizer to boost crop productivity in Ethiopia. The required plant nutrients should be periodically monitored in farming systems.

## Introduction

Maintaining soil resources while meeting the food demand of the growing population is an unsolved problem [1]. Chemical fertilizer has played a major role in global food production over the past 60 years to feed the ever-increasing population. The current challenge for the Ethiopian agricultural sector is low productivity due to a high level of nutrient mining, low use of external inputs, and limited capacity to respond to environmental shocks [2–5]. Cropping areas are subject to severe nutrient losses due to soil erosion and removing dung and crop residue for fuel [6]. Nutrient balance studies by Stoorvogel and Smaling [7] Ethiopia is among the countries with the highest rates of net nutrient losses. Ethiopia is among the countries with the highest rates of net nutrient losses. The annual nutrient deficit is estimated to be negative at 41 kg N, 6 kg P, and 26 kg K per year ha^-1^ in Ethiopian agricultural soil [8–10]. Moreover, the nutrient balance in tef cropping systems is 28 kg N ha^-1^ [11].

The maintenance of soil health depends on balanced fertilization, which includes the application of all the required plant nutrients in proper amounts and forms [12]. Therefore, site-specific nutrient management considering spatial and temporal soil variabilities, crop nutrient requirements, and cropping systems is the most system for achieving targeted goals [2]. This leads to the use of appropriate fertilization, which helps to increase the resilience of crops and therefore plays an important role in climate change adaptation. The application of more and different chemical fertilizers to farmland achieves higher output and productivity [12] through the application of selected nutrient types of crops. Crop plants can only convert approximately 33% of the applied N [13] and 10–15% of the applied P [14] to useful food products (grain).

Tef is a C4 annual small-grain crop native to Ethiopia, known for it’s a resilience [15]. Because, it has grown under different environmental stresses in many parts of Ethiopia. Tef is also a gluten and gluten-like protein-free stable food crop [16]. Tef grains serve as a daily protein source for two-thirds of the Ethiopian population [17]. Thus, fertilizer application has significantly increases crop yields [18]. Approximately 70 to 80% of the inorganic fertilizer purchased by smallholders is known to be applied to tef [19]. Although fertilizer use in Ethiopia has increased notably since 1990, there has been no concomitant yield increase [20]. Low soil fertility and soil acidity [3, 21] inappropriate use of fertilizer and low fertilizer use efficiency [22], inappropriate tillage, and climate variability [23] have limited the low productivity of tef yield.

Nitrogen and P fertilizers have commonly been used in tef production for many years [24–26]. Previous blanket fertilizer recommendations have often applied uniformly across different environmental conditions without considering agro-ecological differences or soil variability considered agro-ecological differences or soil variability [27–29]. Despite substantial increases in fertilizer use in the country [20], deficiencies in N, P, K, S, B, and Zn have been reported in most Ethiopian soils [30–32], and Cu, Mn, and Fe are also deficient in some soils of sub-Saharan Africa [33]. The increased use of mineral fertilizers is considered to be essential for closing yield gaps [34, 35], however, applying unbalanced fertilizer applications over many years limits the potential to maximize and sustain profits. Over the past five decades, numerous fertilizer trials on various crop types have provided empirical evidence confirming that N and P are critical nutrients that need to be supplied to crops in Ethiopia [36, 37]. Some researchers recently argued that KSZnB nutrients are also deficient, with concrete evidence suggesting that other nutrients may not be limiting factors.

Currently, fertilizers containing N, P, S, Zn, and B are imported and distributed in Ethiopia by the Ministry of Agriculture. To shift national fertilizer and import policies, it is essential to identify and confirm the right fertilizer sources for the farming system. Developing site- and crop-specific nutrient recommendations that account for different agroecological zones, and soil types and socioeconomic variability of farmers is now a prerequisite for sustainable crop production and profitability in Ethiopian agriculture [28, 29]. Selecting the right types of fertilizer and applying nutrients in a balanced at appropriate rates tailored to the local climate conditions and soil types is crucial for maximizing tef yield [38]. Although yield responses to different soil nutrient types have been studied in some parts of Ethiopia, they have not been well studied across different soil types and locations in Northwestern Ethiopia.

Many efforts have been made to improve the productivity of tef during the rainy season, although continuous work is necessary. Nevertheless, less emphasis has been given to its production under irrigation, and resulting in a lack of fertilizer recommendations. Recently, successful tef production under irrigation has begun in the Fogera and Mecha districts and surrounding areas of the Amhara region, with yields as high as 4000 kg ha^-1^ recorded through breeding. These represents a substantial potential to fill the yield gap in the country through irrigation. Therefore, the second sets of experiment was designed to assess the contribution of different nutrient types to enhancing the productivity and profitability of tef production under irrigation systems. The hypothesis of this study is that identifying the right nutrient types can reduce yield gaps of tef in both production seasons. Thus, the objective of these two sets of experiments was to investigate the response of tef to different applied nutrients in nitisols and vertisols during rainy and irrigation production seasons in Northwestern Ethiopia.

## Materials and methods

### Descriptions of study area

#### Geographical location

Multiple nutrient omission trials were conducted on farmers’ fields on vertisols and nitisols in northwestern Ethiopia. Two sets of experiments were conducted across two production seasons on different soil types. The first experiment set was performed under rain-fed conditions on two distinct soil types. In contrast, the second set of experiment was carried out under irrigated conditions, exclusively on nitisols. These experiments were conducted at 12 sites across three districts during the rainy season (May to December, 2021/22) and irrigation season (January to May, 2022). The first set of experiment was performed in districts including Yilmana Densa, Gonji Kolela, and Huleteju Enebse, Amhara region, Ethiopia during the rainy season. The second sets of experiment was implemented at two farmers’ fields in the Koga irrigation scheme of Mecha district, Amhara region, Ethiopia during the irrigation production season (2022).

#### Climate conditions and farming systems

**Fig 1:**
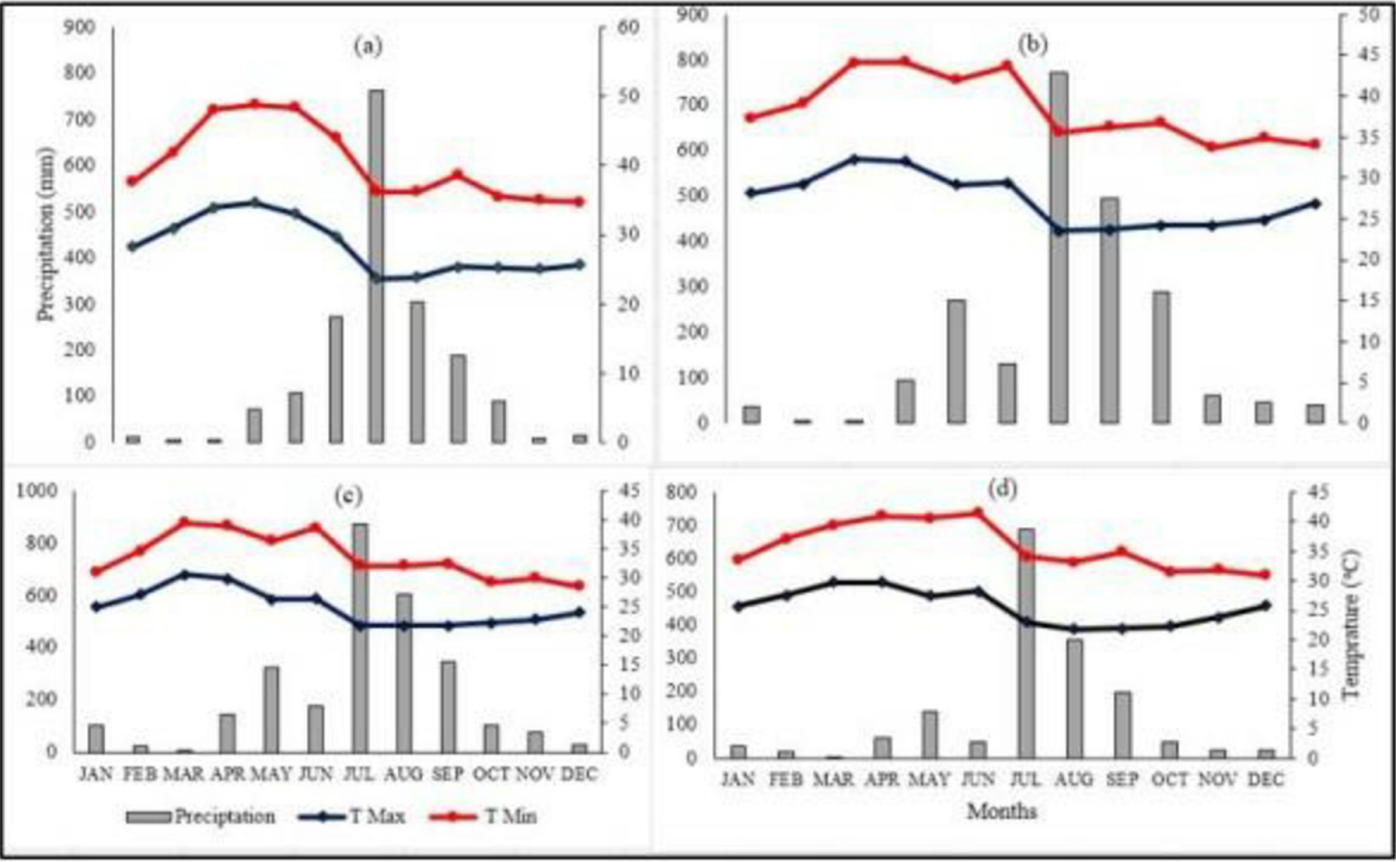
Climate variabilities of the study area (a) Mecha, (b) Yilmana Densa, (c) Gonji Kolela, and Huleteju Enese districts.

The study area falls within the Woyna Dega agro-climatic zone, characterized by an extended dry season lasting from October to May. The rainy season (summer) covers from June to September, with a unimodal rainfall pattern where the greatest amount are occurred from June to August. The total rainfall observed in the districts of Huleteju Enebse, Yilmana Densa, and Gonji Kolela is 1652, 2241, and 2816 mm, respectively, with highest amounts of 689, 771, and 871 mm recorded in July. The mean monthly maximum temperature across the study locations ranges between 22.75°C and 34.6°C, while the mean monthly minimum temperature varies from 5.23 to 15.2°C. Variations in rainfall as well as minimum and maximum temperatures, are observed in experimental areas (Fig 1).

These locations represent the major tef growing areas of the western and eastern Gojjam Ethiopian highlands. Cereal-based cropping systems are the dominant type of farming system in the study area. Maize, tef, and finger millet are the dominant cereal crops grown in the study areas. Bread wheat is also a dominant crop in the Huleteju Enebse district. There are two cereal crop production seasons in study areas. The first or main production season is the rainy season, which spans from May to December. The second production season is the irrigation season, which mostly lasts from January to April.

### Soil characteristics of the study area

Both vertisols and nitisols are the major soil types in the study sites. The experiments were conducted on nitisols in all districts, whereas they were also performed on vertisols in both the Yilmana Densa and Gonji Kolela districts. In the experimental locations, the pH values of vertisols are varied between 6.0 and 6.9. The pH of nitisols ranges from 5.1 to 5.9. In the top 20 cm of soil, the nitisols and vertisols had the greatest and lowest mean soil pH values, 5.13 and 6.49, respectively (Table 1). In the study areas, the vertisols have slightly acidic nature, whereas the nitisols have a considerably acidic [39]. These findings demonstrated that nitisols had a higher acidity than vertisols. The total N content of the soil at the experimental sites varied from 0.06 to 0.14% and from 0.08 to 0.18% in the nitisols and vertisols, respectively (Table 1). According to the rating by Tadesse et al. [39] the soil has low to medium N contents. Relatively vertisols have lower N contents, which is associated with greater N loss in nature. According to the results of Tadesse et al. [39] soil N ranged from low to medium in the study area. The organic carbon content of the soil is observed between 0.7 and 1.3% for vertisols and between 1.0 and 2.2% for nitisols. The available P content of the soil at the experimental site ranged from 4.3 to 5.7 and 5.3 to 14.8 mg kg^-1^in the nitisols and vertisols, respectively. However, the available P content in the soil at the trial sites in the irrigation command area ranged from 7.2 to 35.4 mg kg^-1^. The cation exchange capacity of both soils ranges from high to very high according to the rating Ladon [40]. The available P content of the soil of the experimental site lies within a range of deficiencies for vertisols. It ranged from medium-high for the nitisols in the study area [41]. However, the available p content of the soil at the trial sites in the irrigation command area ranged from medium to high. This may be related to the P fertilization that occurred during both production seasons. This indicates that the addition of chemical fertilizer is needed to improve the tef yield at the study sites.

**Table 1:** Soil parameter descriptive statistics at planting time across sites of study districts during rainy and irrigation production seasons.

Location	pH	OC (%)	P (mg kg^-1^)	CEC	TN (%)	Soil type
Rain-fed						
Site 1	6.86	0.78	5.69	59.20	0.06	Vertisols
Site 2	6.02	0.70	5.69	41.56	0.14	Vertisols
Site 3	6.53	1.01	4.47	45.28	0.13	Vertisols
Site 4	6.53	1.28	4.31	45.32	0.14	Vertisols
Site 5	6.50	1.23	5.41	54.44	0.12	Vertisols
Site 6	5.88	0.95	5.25	39.52	0.14	Nitisols
Site 7	5.42	2.20	9.77	30.04	0.18	Nitisols
Site 8	5.20	2.00	8.95	24.62	0.17	Nitisols
Site 9	5.13	2.16	14.80	32.14	0.08	Nitisols
Site 10	5.21	1.93	12.37	28.70	0.16	Nitisols
Irrigated						
Site 11	5.01	1.51	7.15	-	0.13	Nitisols
Site 12	5.42	1.86	35.74	-	0.14	Nitisols
Mean	5.81	1.47	9.97	40.08	0.13	
SEM	0.19	0.16	2.53	3.59	0.01	
Critical value	5.50	2.00	10.00	30.00	0.20	
Rating	Slightly acid to neutral	Low to medium	Low to high	High to very high	Low to high	
Reference	[39]	[39]	[39]	[40]	[39]	

CEC: cation exchange capacity, N: number of trial sites, P: available P, OC: organic carbon, SEM: standard error of the mean, TN: total N content. The number under parentheses shows the range of values across trial sites.

The Exchangeable potassium (K) of the study area ranges from 0.75 to 1.31 cmol+ kg^-1^ [42–44], which is categorized as high soil K status [40]. In the study areas, the range of values exceeds the critical value of exchangeable K.

### Experimental materials and design

This study was conducted on farmlands across different locations. Two sets of experiments were arranged in a completely randomized design with three replications at each study site. The nutrient omission protocol was used for the experiment. During the rainy production season, the recommended N and P rates were used for nitisols and vertisols in the districts (Table 2). The treatments were composed of one treatment that had six nutrients (N, P, K, S, Zn, and B), followed by the omission of six nutrients, namely: N, P, K, S, Zn, and B. Furthermore, a positive control (recommended NP), negative control (without fertilizer application), and NP+Sx1 treatments were included. Compared with the NP treatment, the NP+Sx1 treatment was used to further evaluate S fertilizer at a rate of 30 kg ha^-1^.

**Table 2:** Rate of applied nutrients in vertisols and nitisols during the rainy season.

No.	Treatment	Nutrient rates (kg ha^-1^) used in the treatment application							
		N		P_2_O_5_		K_2_O	S	Zn	B
		Vertisols	Nitisols	Vertisols	Nitisols				
1	All (NPKSZnB)	80	46	46	69	60	10.5	5	1
2	All–B	80	46	46	69	60	10.5	5	0
3	All–Zn	80	46	46	69	60	10.5	0	1
4	All–S	80	46	46	69	60	0	5	1
5	All–K	80	46	46	69	0	10.5	5	1
6	All–P	80	46	0	0	60	10.5	5	1
7	RNP	80	46	46	69	0	0	0	0
8	No fertilizer	0	0	0	0	0	0	0	0
9	RNP+Sx1	80	46	46	69	0	30	0	0
10	All–N	0	0	46	69	60	10.5	5	1

Note: All nutrients included the application of NPKSZnB, and the rate of NP was determined based on previous recommendations for each location.

A total of nine treatments were also applied for the irrigation production season, which included one treatment with six nutrients (N, P, K, S, Zn, and B), and the other six treatments were included the omission of N, P, K, S, Zn, and B. Moreover, positive control (recommended NP) and negative control (without fertilizer application) treatments were also included. N and P were applied at rates of 92 and 69 kg ha^-1^, respectively, during the irrigation season. The K, S, Zn, and B nutrient application rates were similar to those in the rainy season, as shown in Table 2.

### Experimental management

After preparing the fields, all the sites were broadcasted and row planted for the rainy and irrigation seasons, respectively. The fertilizer and seed rates were calculated without considering furrow spaces for trials under irrigation. The irrigation water was applied to furrows with 40 cm furrow widths at 7–14 day irrigation intervals. All fertilizers were applied by band application at planting except urea which was applied in two splits as top dressing. Nitrogen was applied one month after emergence. Weed management started just after 2 weeks after the emergence of the seeds. Each site was weeded three times during the growing season. All the plants from net plot at the experimental sites were harvested.

### Data collection and analysis

Measurements of yield parameters such as plant height and panicle length were collected from 10 randomly selected plants from the net plot area. Harvesting was performed from the middle 16 rows of 3 m by 3.2 m (9.6 m^2^) net plot area, leaving the border rows as a barrier. Then, the biomass of the plants harvested from the net plot area in the field was measured using a digital balance and converted to kg per hectare. The grain yield was also measured after threshing the biomass collected from the net plot area, after which its weight was measured with a sensitive balance and then converted to kg ha^-1^.

Analyses of variance were executed for grain yield data from each site and combined sites in the districts. A mixed model was used to determine the variation in grain yield with the different types of N, P, K, S, Zn, and B by soil type across the study locations and sites. A fixed model was used to analyze the collected data from each experimental site, whereas mixed models were employed for the combined analysis of multiple sites to estimate the fixed and random effects [45]. In mixed models, the random component specifies that the linear predictor contains a term that randomly varies with a different location that correlates with tef yield. This helps to account for correlation (i.e., observations in a similar location are likely to be more related than observations in other locations and that locations are nested within soil types). The output of the mixed model gives us a list of explanatory values, estimates and confidence intervals of their effect sizes, and p values for each effect. The lme4 and emmeans packages were used for estimation parameters in R programming. The mixed model is explained as follows:

$$y_{ijkl}=\mu +Nut_{i}+Soil_{j}+Rep_{k}+Trt:Soil_{\left(ij\right)}+{\left(1|Location\right)}_{l}+\varepsilon$$ (1)

where μ is the grand mean yield (kg ha^−1^), Nut_i_ is the applied nutrient types (kg ha^−1^)) in the study, the soil type sites were selected based on the World Reference Base Classification, location is the random component, and ϵ is the error term in the mixed model effects.

Various mixed model fits were performed with the help of Akaike’s information criterion (AIC) and the Bayesian information criterion (BIC). Based on this rule, changes in the BICs of 2–6 (the differences between the BICs of the two models) provide weak evidence in favour of more complex models, whereas differences >10 provide strong evidence in favour of more complex models. The Tukey‒Kramer adjustment and its 95% confidence intervals (CIs) were used for statistical estimation. This is because 95% of the CI is a very conservative hypothesis test and ties the uncertainty of sample statistics to the uncertainty measurement. A test of significance for the treatment was performed for significant results as outlined by Cox [46] for situations with heterogeneous variance among treatments. Contrast analysis was also performed between K, S, Zn, and B plus NP and NP alone. Graphs were generated using R programming software.

## Results

### Response of tef grain yield to applied nutrients under rain-fed production system

#### Vertisols

Compared with those of the negative control (without fertilizer application) and All-N treatments, the grain yield significantly (p ≤ 0.001) varied with respect to the applied nutrients in vertisols during the rainy season (Table 3). The grain yield was also significantly (p ≤ 0.001) affected by the recommended N and P compared to that in the no fertilizer treatment and All-N treatments. Compared with treatments that received all six nutrient types (NPSBZnK), certain sites had significantly (p ≤ 0.01) greater yields (Table 3). A significant (p ≤ 0.01) difference in grain yield was detected between All-P and NP only at sites 5 and 6. The grain yield of tef did not exhibit a significant (p > 0.05) increase when KSZnB was omitted in comparison to the application of NP or NPS nutrients. A greater tef yield was recorded from All-Zn (1712 kg ha^-1^) in the vertisols at site 4 (Table 3). However, the lower yield obtained from the no fertilizer treatment ranged from 253 to 432 kg ha^-1^ across the study sites. Similarly, relatively lower yields ranged from 232 to 804 kg ha^-1^ from N-omitted treatment across the study sites.

**Table 3:** Tef grain yield (kg ha^-1^) response to nutrient types at different sites in vertiols.

Treatments	Site 1	Site 2	Site 3	Site 4	Site 5
All	1440	1629	1264	1508	973
All-B	1438	1695	905	1547	1023
All-Zn	1346	1569	1470	1712	938
All-S	1349	1446	1120	1618	1127
All-K	1215	1675	1190	1473	1147
All-P	1330	1372	1356	1660	369
RNP	1044	1552	1260	1613	1154
NF	432	418	209	398	253
RNP+Sx1	1292	1568	1182	1506	986
All-N	547	490	232	804	312
LSD (0.05)	287	354	277	337	156
CV (%)	14.6	15.4	15.9	14.2	11.0
SEM	±71.4	±91.5	±83.1	±81.2	±66.8
p	***	***	***	***	***

All = NPKSZnB, CV = coefficient of variance, LSD = least significant difference, NF = no fertilizer, p: significance level, RNP = recommended N and P, RNP+Sx1 = recommended N and P with the addition of 30 kg ha^-1^ S, SEM = standard error of the mean

***: highly significant at <0.0001%.

### Nitiosls

Grain yield was significantly (p ≤ 0.01 and 0.001) different from the nutrients applied in nitiosols during the rainy season (Table 4). Additionally, the application of N and P had a substantial (p < 0.001) effect on grain yield as compared to the All-N and no fertilizer treatments. In comparison to treatments that received all six nutrient types (NPSBZnK), several sites produced considerably higher yields (p < 0.01) (Table 3). Only at sites 5 and 6, grain yield was a significant (p < 0.01) difference between All-P and NP found. When KSZnB nutrients were not applied, the grain yield of tef did not increase significantly (p > 0.05) as compared to when NP or NPS nutrients were applied. A higher tef yield was recorded from the application of All-B (1652 kg ha^-1^) in the nitisols at site 10 (Table 4). However, across all study sites, the reduced yield from the no fertilizer treatment varied from 440 to 571 kg ha^-1^. Similarly, across all research sites, the N-omitted treatment produced comparatively lower yields ranging from 543 to 728 kg ha^-1^. Both the unfertilized and All-N treatments showed lower grain production at site 9 compared to all other sites.

**Table 4:** Tef grain yield (kg ha^-1^) response to nutrient types at different sites in nitisols.

Treatments	Site 6	Site 7	Site 8	Site 9	Site 10
All	933	1141	1039	1323	1443
All-B	1078	1209	1088	1179	1652
All-Zn	1065	1138	957	1258	1328
All-S	1104	1110	1011	1138	1415
All-K	1076	1231	887	1198	1393
All-P	829	1030	1013	1162	1139
RNP	1118	1202	974	1218	1144
NF	571	469	526	440	448
RNP+Sx1	967	1135	1032	1131	1305
All-N	728	663	485	543	557
LSD (0.05)	242	305	221	242	258
CV (%)	15.0	17.4	14.4	13.4	13.0
SEM	±38.9	±52.7	±42.8	±58.0	±72.4
p	**	***	***	***	***

All = NPKSZnB, CV = coefficient of variance, LSD = least significant difference, NF = no fertilizer, p: significance level, RNP = recommended N and P, RNP+Sx1 = recommended N and P with the addition of 30 kg ha^-1^ S, SEM = standard error of the mean

***: highly significant at <0.0001%

** significant at <0.001.

### Tef grain yield response under both soil types

The combined mean grain yield of the tef significantly (p ≤ 0.01) varied with the application of different plant nutrients across sites in both soil types (Table 5). The response to omitted plant nutrients significantly varied with the interaction effect of nutrient type across sites.

**Table 5:** Mean square values of combined ANOVA for tef grain yield on different soil types in the study areas.

No	Source of variations	Df	Nitisols	Vertisols
1	Treatment	9	2455.2***	4487.8***
2	Site	9	1222.5***	134.1***
3	Replication	2	18.6 ^ns^	6.4**
4	Treatment × Site	64	21.2*	84.7***
5	Error	128	27.2	53.5
	Adjusted R^2^	-	0.77	0.85

Df: degree of freedom, ns: non-significant at p>0.05

*: Signiant a p<0.05

**: highly significant at p<0.01

***: highly significant at p<0.001.

**Fig 2:**
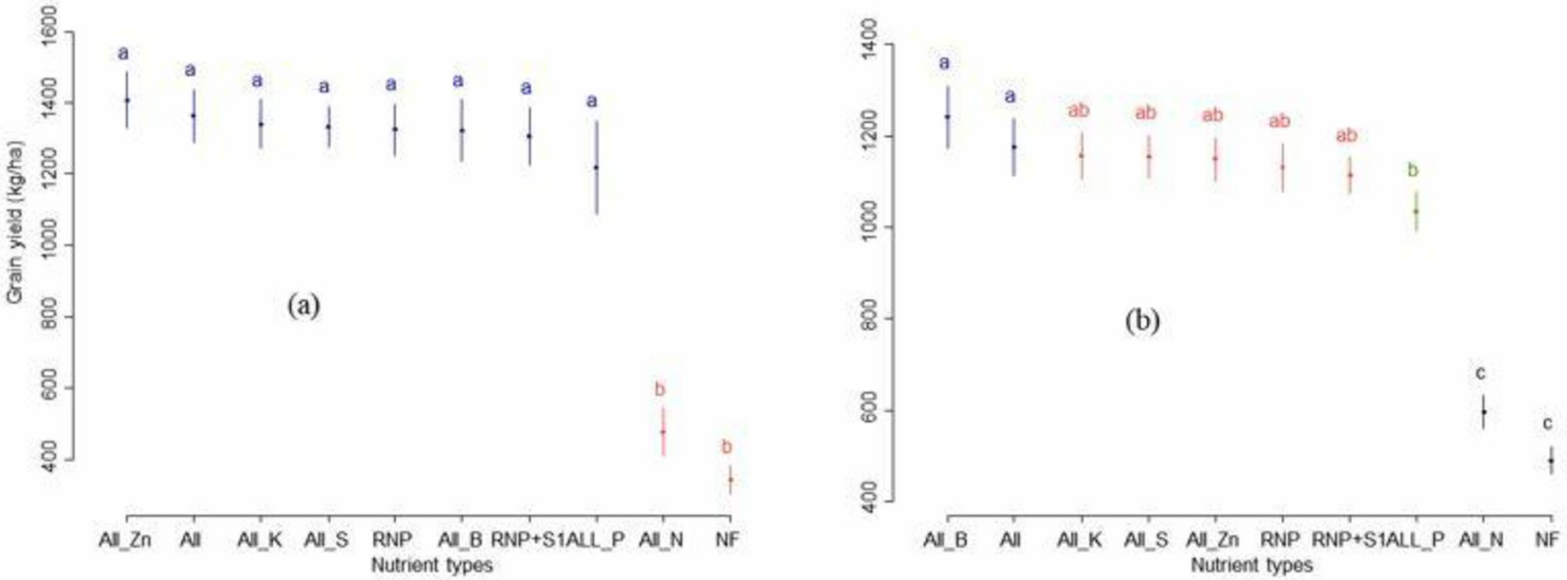
The combined analysis of tef yield response to nutrients in a) vertisols and b) nitisols under rain fed production system. *Short lines at the top of each bar represent the standard error of nutrient types*, *lowercase letters indicate significant differences (p<0*.*05) among treatments*.

**Fig 3:**
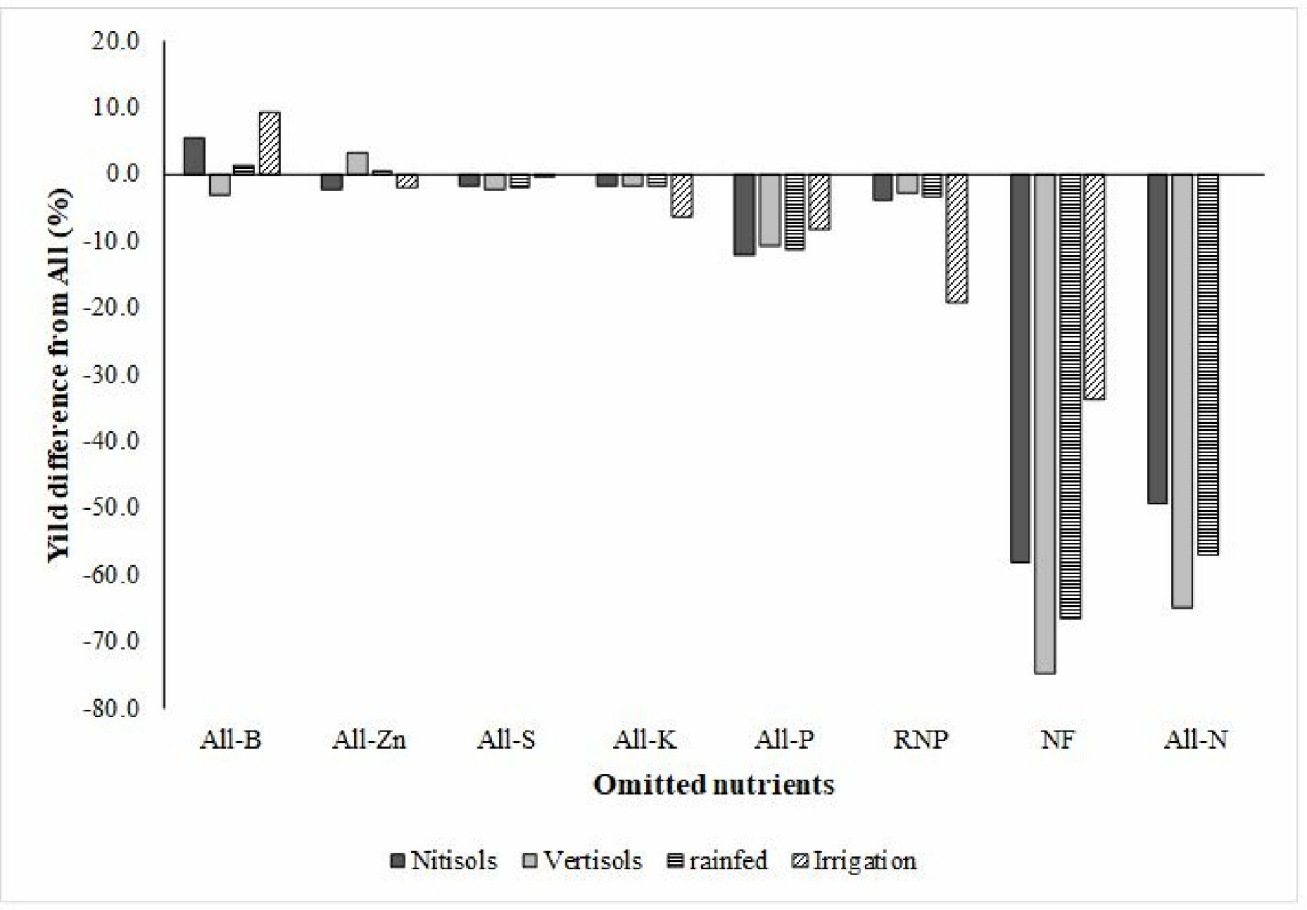
The percentage of yield-limiting nutrients as a function of the response of each nutrient to all applied nutrient types in the study soils under the rain-fed and irrigation production systems.

The mean grain yield significantly (p ≤ 0.001) increased in response to plant nutrient application compared with All-N and the control across all trial sites in the vertisols of the study districts (Fig 2). The figure also shows that the grain yield also significantly (p ≤ 0.001) differed from the omission of nutrients in the nitisols. A significant (p ≤ 0.01) difference in grain yield was observed between the no P treatment and all the other nutrient treatments in the nitisols. Generally, a higher yield was observed for All-Zn and All in vertisols. Nonsignificant (p > 0.05) grain yield differences were observed from the omission of K, S, Zn, and B fertilizers in vertisols and nitisols (Fig 3). The highest (1241 kg ha^-1^) and the lowest (491 kg ha^-1^) mean grain yields were recorded from All-Zn and no fertilizer treatments in nitisols, whereas the maximum (1407 kg ha^-1^) and the minimum (342 kg ha^-1^) mean grain yields were obtained from All-Zn and no fertilizer treatments in vertisols.

### Response of tef yield to nutrient types under irrigation production system

Both grain and biomass yields showed highly significant (p ≤ 0.01) differences among treatments at all experimental sites under the irrigation production system. Table 6 indicates that the application of N and P nutrients at both sites resulted in greater grain yields of 1693 and 1835 kg ha^-1^. The minimum grain yields (1053 and 1151 kg ha^-1^) were observed from no fertilizer treatment. From sites 11 and 12, the N-omitted treatment also contributed grain yields by 1501 and 1184 kg ha^-1^, respectively.

**Table 6:** Tef grain and biomass yields (kg ha^-1^) response to applied nutrients under irrigation, at two sites, in nitisols.

Treatments	Grain yield		Biomass yield	
	Site 11	Site 12	Site 11	Site 12
All	1584.8	1741	5111.1	5486.1
All-B	1659.7	1972.2	5930.6	5486.1
All-Zn	1605.8	1656.9	5601.9	5277.8
All-S	1397.6	1926	5138.9	5486.1
All-K	1512.7	1600.3	4949.1	4791.7
All-P	1079.6	1971.9	4122.2	5694.4
RNP	1693.2	1835.1	6034.7	5555.6
NF	1052.5	1150.7	3402.8	2986.1
All-N	1501.4	1184	5083.3	3263.9
p	**	**	**	**
LSD	308.8	347.6	1248.6	896.7
CV (%)	12.4	12.1	14.4	10.7

All = NPKSZnB, CV = coefficient of variance, LSD = least significant difference, NF = no fertilizer, p: significance level, RNP = recommended N and P

**: significant at 1%.

In the combined analysis, the effects of omitted nutrients on grain and biomass yields were significantly different (p < 0.01) from those of the unfertilized and N-omitted treatments (Table 7). Relatively higher yields were obtained from the All-N treatment and unfertilized treatment under the irrigation system. However, neither the addition nor omission of K, S, Zn, or B had a visible contribution to nor no significant (p > 0.05) effect on either the grain or biomass yield of tef.

**Table 7:** Combined analysis of tef yield (kg ha^-1^) response to applied nutrients types in nitisols of Mecha district during irrigation season.

Treatments	Grain yield	Biomass yield
All	1662.9	5298.6
All-B	1816.0	5708.3
All-Zn	1631.4	5439.8
All-S	1661.8	5312.5
All-K	1556.5	4870.4
All-P	1525.7	4908.3
RNP	1764.1	5795.1
NF	1101.6	3194.4
All-N	1342.7	4173.6
Mean	1562.5	4966.8
p	**	**
LSD	316.3	860.1
CV (%)	17.4	14.9

All = NPKSZnB, CV = coefficient of variance, LSD = least significant difference, NF = no fertilizer, p: significance level, RNP = recommended N and P

**: significant at 1%

A highly significant (p < 0.000) decrease in grain yield (536.1–847.6 kg ha^-1^) was obtained from the omission of N compared to the recommended N and P during the rainy season (Table 8). Similarly, a significant (p = 0.039) reduction in tef grain yield was recorded during the irrigation season. Phosphorus omission reduced the grain yield from 11.6 to 238.4 kg ha^-1^ under both production systems. Without the application of N and P, a highly significant (p < 0.000) grain yield reduction ranging between 640.8 and 958.5 kg ha^-1^ was recorded during both production systems. A decrease in yield from 101.2 to 662.5 kg ha^-1^ was attained due to the omission of different nutrients, except for B, during the irrigation season. Relatively lowest grain yield reduction (-983 kg ha^-1^) was recorded from unfertilized plots in vertisols, followed by N omitted plots under rain- fed season.

**Table 8:** Mean yield difference values (kg ha^-1^) from the contrast analysis of omitted nutrient types with recommended NP nutrient under vertisols and nitisols in rain fed and irrigation system.

S/N	Contrast	Rain fed			Irrigated
		Combined	Vertisols	Nitisols	Nitisols
1	All vs RNP	41.6 [0.947]	38.4 [0.995]	44.7 [0.939]	-101.2 [0.943]
2	All-B vs RNP	53.4 [0.881]	-2.9 [1.000]	109.7 [0.348]	51.8 [0.994]
3	All-K vs RNP	20.6 [0.996]	15.5 [0.999]	25.6 [0.991]	-207.6 [0.586]
4	All-N vs RNP	-691.8 [0.000]	-847.6 [0.000]	-536.1 [0.000]	-421.4 [0.039]
5	All-P vs RNP	-11.6 [0.999]	-107.1 [0.837]	-96.8 [0.478]	-238.4 [0.455]
6	All-S vs RNP	16.0 [0.998]	7.7 [1.000]	24.3 [0.993]	-102.3 [0.941]
7	All-Zn vs RNP	50.3 [0.901]	82.6 [0.928]	18.1 [0.998]	-132.8 [0.870]
8	NF vs RNP	-811.6 [0.000]	-982.5 [0.000]	-640.8 [0.000]	-662.5 [0.000]
9	(RNP+Sx1) vs RNP	17.8 [0.998]	-17.8 [0.999]	17.5 [0.998]	-
	SEM	57.5	104.1	59.6	145.8

SEM: standard error of the means; values under the parentheses show the p values of contrast between NP and other omitted nutrients. The numbers under parentheses show the p values of the significance levels.

### Contribution of nutrients to tef grain yield in both soil types

Fig 3 shows that without N fertilizer, 49% and 65% decreases in yields were observed in the nitisols and vertosols, respectively, during the rainy season. Similarly, the omission of P fertilizer also reduced the grain yield of the tef by 12 and 10% in the nitisols and vertisols, respectively. The omission of both N and P diminishes grain yield by 19% under irrigation. However, there was no significant variation due to the omission of different nutrients; the omission of B improved the tef yield by 5.5% in the nitisols, whereas Zn omission increased the tef grain yield by 3.2% in the vertisols. Below 2%, nonsignificant yield decreases were observed when K and S were omitted. Unfertilized treatment reduced the grain yield by 34 and 67% in the irrigation and rainfed production systems, respectively. A nonsignificant less than 10% yield reduction was observed when P, K, S, and Zn fertilizers were omitted from the irrigation production system. However, the omission of B increased the tef yield by 9% in the nitisols during the irrigation season.

### Relationship between applied nutrients and biological parameters

**Fig 4:**
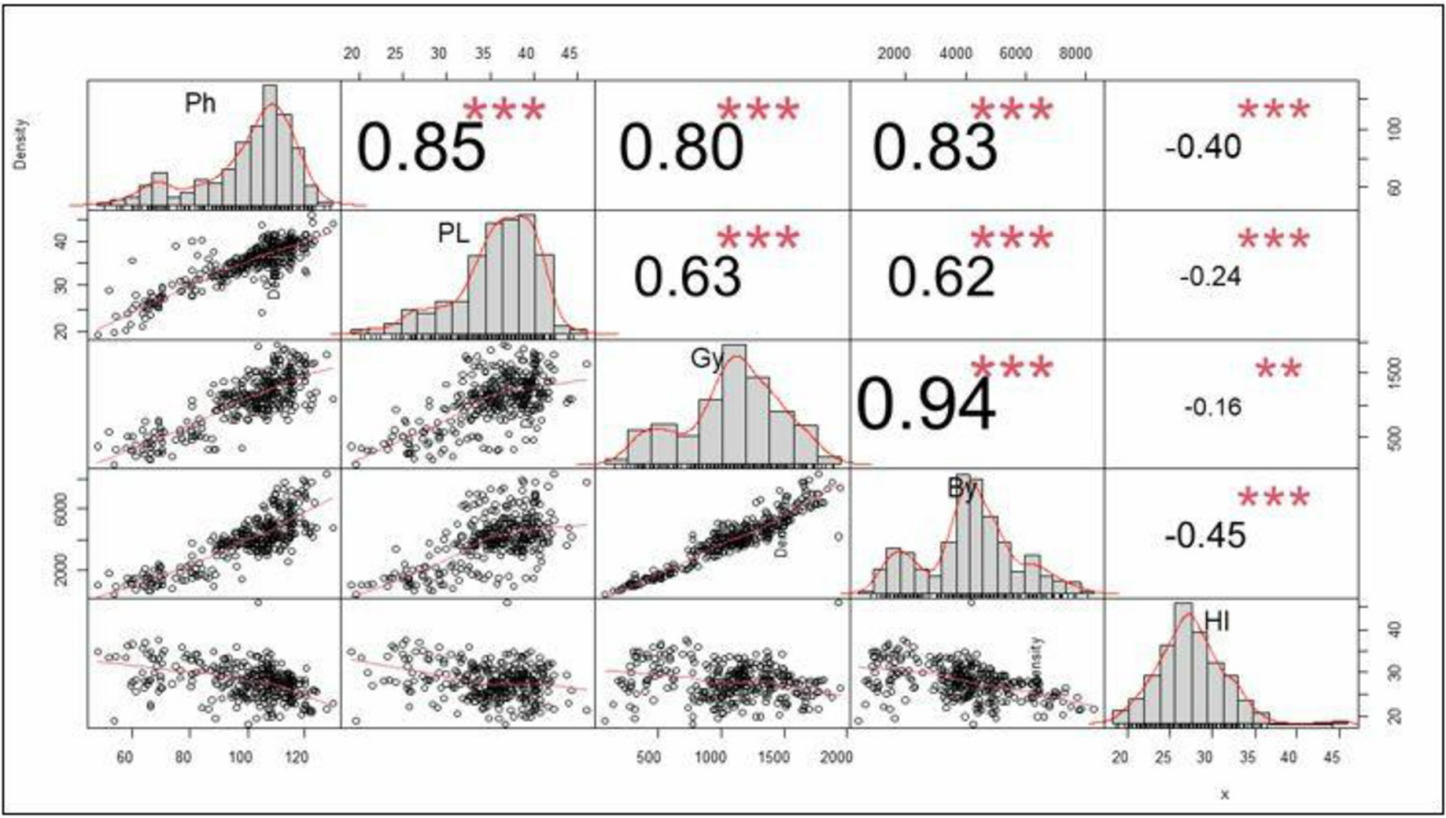
Correlation of yield and yield components of tef for applied nutrient types across sites in North West Amhara. Ph: plant height, PL: panicle length, Gy: grain yield, By: biomass yield; HI: harvest index, **: significant at 5% and *** significant at 1%. The numbers in the figure indicate the correlation coefficient values.

With respect to the different applied fertilizers, the grain yield was significantly and positively correlated with biomass (r = 0.94***), plant height (r = 0.80***), and panicle length (r = 0.63***), whereas it was inversely and significantly correlated with the harvest index, with a correlation coefficient of 0.46***, and the 1000-seed weight, with r = -0.45 at p < 0.001 (Fig 4).

## Discussion

### The contribution of different nutrients to tef yield in nitisols and vertisols (rainy season)

The significant difference in grain yield resulted from the omission of nutrients compared to those in the negative control (without fertilizer application) and All-N treatments during the rainy season. Our findings disagree with those of Gebrehawariyat et al. [47] who suggested that applying K fertilizer increases tef production. As we can see from our study, the majority of plant nutrients (KSZnB) come from the soil, but the soil does not provide an adequate amount of all the nutrients, particularly the N and P that plants need in the proper amounts. Nitrogen is the most yield-limiting nutrient in most soils, agroecosystems, and regions [43]. It is also a universal yield-limiting nutrient [48]. Therefore, the use of synthetic N fertilizers accounts for the increase in food consumption worldwide. Different studies indicate that crops grieve severe N deficits and reduced yields as a result of inadequate N and other nutrients [49]. This might be associated with the severe erosion of applied NP fertilizer and native soil nutrients in agricultural fields [8]. Our research contradicted the assertion of Habte and Boke [50] who reported that NPS increased tef yield. Furthermore, our findings disagree with those of Kihara et al. [33], who claimed that African soils are deficient in micronutrients. The biological yield results were directly correlated with the locations where our soil analysis revealed low N and P concentrations at the trial sites. Thus, fertilization is mandatory to address these deficiencies. A greater yield of All-Zn and All nutrients was observed in the vertisols. The All-N treatment had a lower yield following the negative control for both soil types. The production of tef is not limited by the absence of P nutrient application in vertisols. This result contradicts the findings of Alemayehu et al. [43], who reported that P limits the productivity of tef in vertisols.

The absence of KSZnB nutrients resulted in nearly comparable grain yields with the only application of recommended NP and All fertilizers in both soil types during rain-fed production season. Currently, the soil can supply these nutrients without limiting them [36, 43, 51, 52]. Selassie et al. [52] also demonstrated that K fertilizer cannot increase agricultural crop productivity in northwestern Ethiopia, which supports our findings. However, the result differed from the previous findings, which show Ethiopian soils are deficient in macronutrients (KS) and some micronutrients. Hailu et al. [31] and [32, 53] reported that the application of blended NPS and K fertilizer increases crop productivity in southern Ethiopia. However, this report did not support our findings. Brhane et al. [54] reported that the application of K fertilizer boosts the yield of cereal crops at vertisols in the northern part of Ethiopia, which disagrees with our findings.

### Response of tef to nutrient types under irrigation production system

The omission of N diminishes grain and biomass yields under irrigation. The control (without fertilizer) treatment grain and biomass yields also declined. Phosphorus was not a limiting nutrient in tef productivity under the Koga irrigation scheme, Mecha district. The grain yield result is directly correlated as the before-planting soil result where the available P content of the soil was high. This might be associated with the accumulation of residual P due to the greater application of P fertilizer under two production seasons (rain-fed and irrigation).

The omission of N, as well as the control treatments (no fertilizer), significantly affected grain and biomass yields. The different soil characteristics of the trial sites showed variable responses to grain and biomass yield. This indicated that N nutrients play a major role in determining the biological yield of irrigation systems. This finding is similar to the results of main season tef production for nutrient omission experiments reported by Bazie et al. [21]; Amare et al. [36], Alemayehu et al. [43], Acharya et al. [55]; Chala et al. [51] who described as N, is the greatest limiting nutrient for crop productivity. Similar to the individual experimental sites, the combined statistical analysis result showed that both grain and biomass yields of tef significantly differed among the treatment methods. Automatic responses of both the grain and biomass yields of tef were observed when either of the two or both major nutrients (NP) were omitted. The two nutrients strongly affect tef productivity and have a clear potential influence on limiting tef biological yields under irrigation systems, which is in line with the findings of Amare et al. [36], who reported NP nutrients are important nutrients compared to other plant nutrients in northwestern Ethiopia.

Relatively high grain yields were observed in the All-N and unfertilized treatments during the rainy season. In particular, the yield without N was nearly equal to the yield in the control treatment, even if all other nutrients were applied at optimal levels during the irrigation season. Thus, N is the governing production input in Ethiopian soils. According to the combined analysis results, N was still the leading yield-limiting nutrient in the study district, followed by P, which is in line with the results reported by Amare et al. [36], who indicated that low N and P availability in soils for cereal production in the Mecha district where Koga irrigation scheme is found.

The omissions of P, K, S, and Zn nutrients did not show a significant yield variations during the irrigation production system. However, the omission of B increased the tef yield in nitisols during the irrigation season. This result is similar to the results of nitisols during the rainy season. This shows that N is also the major nutrient required for tef under irrigation. Our finding is in line with the results found under the main season crop production system in the region.

### Response of tef yield for each nutrient

Nitrogen deficiency was observed in both soil types under rain-fed and irrigation production systems. This finding agrees with many research reports revealing that N is the first and most limiting nutrient for tef production in Vertisols of Ethiopia [36, 43, 56]. The reduced yield resulting from the omission of N fertilizer may be linked to a lack of plant nutrients in the native soil, particularly low levels of N and P. Correcting N deficiency can be achieved through approprite N fertilization and soil management practices [57]. Nevertheless, between only 30 and 50% of the applied N fertilization is typically taken up by cereal crops [13]. Future research is required to enhance N fertilization efficiency through different technologies. Gollobally, 57% of total fertilizer consumption is for N, followed by P (24%), to increase grain yield [58]. This indicates the importance of N as the most significant key nutrient in the world. However, our finding diferr from those of Heffer et al. [58] who reported that K (19%) fertilizers are primarly used to improve crop quality. Therefore, the timely application of N is essential to sustain yield and biomass production.

Nutrient deficiency, particularly N and P, is a serious threat to Ethiopian soils [31, 59]. It is also an important critical yield-limiting nutrient for agricultural crop production worldwide, particularly in the tropics, because of fluctuations in temperature and precipitation [57, 60]. According to EthioSIS [61] widespread multi-nutrient deficiencies and deteriorating soil health are the cause of low nutrient use efficiency, productivity, and profitability. Therefore, the appropriate use of yield-limiting nutrients in crop production is crucial for increasing crop yield and quality, environmental safety, and economic considerations [48].

Phosphorus is the second essential plant nutrient for global food production [14]. However, only 10 to 15% of applied P fertilizer is taken by crops, which indicates that P fertilizer must be applied to feed the ever-increasing population. Much of the residual P fertilizer remains unavailable to crops due to its conversion into non-usable forms. To meet the productivity and profitability goals of food crop production, 4R nutrient stewardship strategies are critically important [62]. Thus, optimizing nutrient management practices and technologies is required for agricultural crop production systems, both under rain-fed and irrigation seasons.

The omission of B did not show a regular trend in tef yield across both soil types. This suggests that Zn in vertisols and B in nitisols are adequately available in soil nutrient stock. The non-significant yield decrease observed with the omission of K and S fertilizer shows that the indigenous soil is capable of supplying these nutrients.

### Association of tef biological parameters to applied nutrients

The grain yield showed a significant and positive correlation with yield-related parameters when different nutrient types were applied. Generally, fertilizer application and crop yield have a strong positive relationship. In the tef fertilizer study, aboveground biomass, plant height, and panicle length are the most crucial yield attributes parameters contributing to grain production [26, 63]. Similar findings were reported by Alemayehu et al. [43], who indicated tef yield and applied nutrients significantly influenced yield component parameters.

### Conclusions and recommendations

In this study, a significant biological yield advantage was recorded from the recommended applications of N and P. However, the yield did not improve with the application of K, S, Zn, and B nutrients. The yield obtained from the N-omitted treatment was equivalent to that from from the no fertilizer-added (negative control) treatment, despite all other nutrients being applied at optimal levels at the N-omitted treatment. This infers that N is the first and major limiting nutrient for tef production in the study area’s soil types under both rain-fed and irrigation production systems. Phosphorus was found to be the second most common yield limiting nutrient, mostly in nitisols under rain-fed production. Potassium, S, Zn, and B were not mandatory for tef production.

The study implies that N and P are key nutrient to enhance tef yield productivity for smallholder farmers. Further research on the N and P fertilizer rates is suggested to determine the optimum production curve. Periodical assessment of nutrient status of agricultural soil mayalso be necessary for tef production in the future.

## Supporting information

S1 Appendix (DOCX)

S1 Data (XLSX)
