# Supplementary material for: Response of tef (Eragrostis tef (Zucc.) Trotter) yield to nutrient management under rainy and irrigation production systems in northwestern Amhara, Ethiopia
Source: PLoS One. 2025 Jan 31;20(1):e0315730. doi: 10.1371/journal.pone.0315730 (PMC11785318; doi:10.1371/journal.pone.0315730)
Supplement: S1 Appendix — (DOCX) [file pone.0315730.s001.docx]

**Appendix**

Table 1. Response of biomass yield and yield related parametrs to nutrients

| Treatments | By (kgha^-1^) | Ph (cm) | PL (cm) | HI |
| --- | --- | --- | --- | --- |
|  | Vertisols |  |  |  |
| All | 5368 | 113 | 38 | 26 |
| All-B | 5099 | 113 | 39 | 26 |
| All-Zn | 5338 | 108 | 37 | 26 |
| All-S | 5130 | 114 | 38 | 26 |
| All-K | 5455 | 114 | 38 | 25 |
| All-P | 4828 | 107 | 37 | 25 |
| RNP | 5418 | 114 | 38 | 25 |
| NF | 1180 | 70 | 27 | 28 |
| RNP+S_1_ | 5306 | 114 | 37 | 25 |
| All-N | 1513 | 73 | 29 | 31 |
| LSD (0.05) | 892.1 | 5.8 | 2.3 | 2.2 |
| CV | 16.2 | 7.0 | 9.3 | 8.5 |
| p | *** | *** | *** | *** |
| Nitisols | | | | |
| All | 3952 | 101 | 37 | 28 |
| All-B | 3909 | 99 | 36 | 29 |
| All-Zn | 3853 | 99 | 35 | 29 |
| All-S | 3753 | 98 | 36 | 29 |
| All-K | 3901 | 101 | 37 | 28 |
| All-P | 3582 | 98 | 36 | 28 |
| RNP | 3949 | 99 | 35 | 28 |
| NF | 1589 | 69 | 27 | 32 |
| RNP+S_1_ | 3842 | 99 | 36 | 28 |
| All-N | 1839 | 69 | 27 | 33 |
| LSD (0.05) | 368.7 | 6.0 | 2.6 | 2.9 |
| CV | 15.5 | 5.5 | 6.3 | 8.2 |
| p | *** | *** | *** | ** |
